# 2198: Gender differences in the pharmacology of buprenorphine sublingual tablets in Hispanics/Latinos: An underrepresented population

**DOI:** 10.1017/cts.2017.119

**Published:** 2018-05-10

**Authors:** Darlene Ivelisse Santiago, Jorge Duconge

**Affiliations:** University of Puerto Rico-Medical Sciences Campus, San Juan, Puerto Rico

## Abstract

OBJECTIVES/SPECIFIC AIMS: The objective of this study is the pharmacology of sublingual Buprenorphine in Hispanics/Latino men and women. Specifically we plan to: (1) Administer sublingual buprenorphine to Hispanic/Latino men and women volunteers, and measure the circulating amounts of the drug in the bloodstream as a function of time; that is, pharmacokinetics of buprenorphine. The goal of the proposed study is to evidence that there are gender and ethnic differences in the pharmacokinetics of sublingual buprenorphine between not only Hispanics/Latinos and non-Hispanics/Latinos (Caucasian), but also within Hispanic/Latino men and women. METHODS/STUDY POPULATION: We are proposing a phase 1 of buprenorphine using 12 healthy volunteers. To test for differences in pharmacokinetics between Hispanic/Latino men and women, 6 Hispanic/Latino men, and 6 Hispanic/Latino women 21 years of age and older will be recruited. The volunteers should be living in Puerto Rico, and must have both parents born in Puerto Rico. Sublingual buprenorphine will be administered using a low dose of 16 mg one time only. Blood samples will be collected from each volunteer at *t*=0, 1, 2, 4, 6, 8, 12, and 24 hours after administration. The amount of circulating drug in the bloodstream of the volunteers will be measured using liquid chromatography combined with mass spectrometry. Pharmacokinetic obtained parameters will be maximal plasma concentration, minimal plasma concentration, predose concentration, 24 hour post predose concentration, the time for maximum concentration. The area under the curve will be determined by the trapezoidal rule. Male Versus female data will be compared using 2-tailed *t*-test. RESULTS/ANTICIPATED RESULTS: We anticipate that: (1) Hispanic/Latino women will have longer circulating times of the drug in the bloodstream and higher maximum concentrations, compared with men. (2) Hispanic/Latino men and women will have higher amounts of the circulating drug, compared with already reported pharmacokinetic data of non-Hispanic Caucasian men. DISCUSSION/SIGNIFICANCE OF IMPACT: Gender differences have been elucidated in the prevalence rates of substance abuse, health service utilization, treatment outcomes, and physiological consequences of drug consumption in the United States. It is known that in general, women progress from drug use to dependence must faster than men; women also suffer more severe physical and emotional consequences than men, yet women seek treatment for drug addiction in lower rates compared with men. Women also show lower pharmacological treatment effectiveness as they are less likely to feel satisfied upon entering a substance abuse treatment and they show higher cravings. Sublingual buprenorphine is a very popular and relatively new medication used primarily for opiate addiction since 2002. Gender differences have been elucidated in the pharmacology of buprenorphine sublingual tablets used for the treatment of opioid addiction. One study showed that women had higher concentrations of circulating parent drug and it is metabolites compared with men. One metabolite in particular norbuprenorphine was found in almost double the plasma concentration in women. Interestingly, gender differences were not pursued at all by the Pharmaceutical Company sponsoring the approval of the sublingual Buprenorphine by the FDA. The cytochrome enzyme CYP 3A4 responsible for the metabolism of Buprenorphine has higher activity in Caucasian/African American women compared with men. However these studies failed to design and recruit significant amount of patients with Hispanic ethnicity to adequately elucidate the gender differences within this ethnic group. Higher plasma concentrations and longer circulation times of a drug may result not only in lower efficacy outcomes but also higher toxicity and undesired effects. Unfortunately, the lack of pharmacological effectiveness and lack of satisfaction in women undergoing drug treatment programs has not been adequately studied to understand the gender difference in pharmacological treatment outcomes between Hispanic/Latino men and women. Due to the under-representation of Hispanic/Latino men but most importantly women in s studying the pharmacology of sublingual Buprenorphine, and considering the well-established gender difference of the principal enzyme (CYP 3A4) responsible for the pharmacology of Buprenorphine, we are proposing a pilot study of the pharmacology of sublingual Buprenorphine in Hispanic/Latino volunteers living in Puerto Rico with equal number of male and female patients. We expect our research to clinically and scientifically elucidate the gender differences of sublingual buprenorphine for opioid addiction in Hispanics/Latinos. The outcome of such research will be the foundation of subsequent clinical studies that aim in updating the current standard of care for Hispanic/Latino men and women that require therapy for opioid addiction.

